# Supplementary material for: Surface Microbiome Profiling of Dental Elevators Using Third‐Generation Sequencing: Implications for Infection Control in Dental Practice
Source: Microbiologyopen. 2025 Dec 8;14(6):e70178. doi: 10.1002/mbo3.70178 (PMC12685768; doi:10.1002/mbo3.70178)
Supplement: Supplementary file 1 — customer_backup. [file MBO3-14-e70178-s001.zip › customer_backup/customer_backup/function_analysis/BugBase/treat/otu_contributions/order/taxa_legend.pdf]

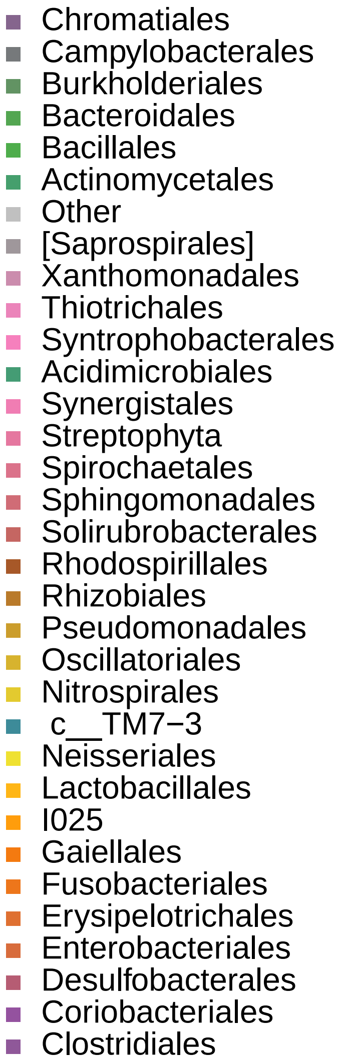
Chromatiales
Campylobacterales
Burkholderiales
Bacteroidales
Bacillales
Actinomycetales
Other
[Saprospirales]
Xanthomonadales
Thiotrichales
Syntrophobacterales
Acidimicrobiales
Synergistales
Streptophyta
Spirochaetales
Sphingomonadales
Solirubrobacterales
Rhodospirillales
Rhizobiales
Pseudomonadales
Oscillatoriales
Nitrospirales
c__TM7−3
Neisseriales
Lactobacillales
I025
Gaiellales
Fusobacteriales
Erysipelotrichales
Enterobacteriales
Desulfobacterales
Coriobacteriales
Clostridiales
